# Complete Genome Sequence of *Pseudomonas chilensis* Strain ABC1, Isolated from Soil

**DOI:** 10.1128/MRA.00775-20

**Published:** 2020-09-24

**Authors:** Daniel Valenzuela-Heredia, Carlos Henríquez-Castillo, Raúl Donoso, Paris Lavín, María S. Pavlov, Oscar Franchi, José Luis Campos

**Affiliations:** a Facultad de Ingeniería y Ciencias, Universidad Adolfo Ibáñez, Viña del Mar, Chile; b Laboratorio de Fisiología y Genética Marina (FIGEMA), Centro de Estudios Avanzados de Zonas Áridas (CEAZA), Coquimbo, Chile; c Facultad de Ciencias del Mar, Universidad Catolica del Norte, Coquimbo, Chile; d Programa Institucional de Fomento a la Investigación, Desarrollo, e Innovación, Universidad Tecnológica Metropolitana, Santiago, Chile; e Departamento de Biotecnología, Facultad de Ciencias del Mar y Recursos Biológicos, Universidad de Antofagasta, Antofagasta, Chile; f Laboratorio de Complejidad Microbiana y Ecología Funcional, Instituto Antofagasta, Universidad de Antofagasta, Antofagasta, Chile; g Pontificia Universidad Católica de Valparaíso, Valparaíso, Chile; University of Arizona

## Abstract

Here, we report the complete genome sequence of *Pseudomonas chilensis* strain ABC1, which was isolated from a soil interstitial water sample collected at the University Adolfo Ibañez, Valparaiso, Chile. We assembled PacBio reads into a single closed contig with 209× mean coverage, yielding a 4,035,896-bp sequence with 62% GC content and 3,555 predicted genes.

## ANNOUNCEMENT

*Pseudomonas* is a widespread genus of Gram-negative bacteria belonging to the *Gammaproteobacteria*, inhabiting a great diversity of environments (1). This variability of habitats is reflected in the diversity of structure and composition of *Pseudomonas* species genomes. We report here the complete genome sequence of *Pseudomonas chilensis* strain ABC1, isolated from a soil interstitial water sample of a coastal environment with a Mediterranean climate influenced by the Humboldt current. The genomic information reveals potential bioremediation features of the ABC1 strain, supported by the presence of genes involved in resistance against metals and the synthesis of metabolites that promote plant growth (2).

The strain ABC1 is an aerobic fluorescent species that grows in acetate (as the sole carbon and energy source) between 15°C and 30°C. The strain was isolated from soil interstitial water (0 to 5 cm deep) in Valparaiso, Chile (33.035021S, 71.595079W), using a liquid preculture on AAM medium (3) for 10 days, followed by plating onto AAM agar. A single colony was transferred onto a sterile AAM agar plate and then incubated at 20°C for 72 h. A sample was cryopreserved at −165°C. The taxonomic identity of the strain was confirmed by partial sequencing of the 16S rRNA gene. PCR was performed using the 27F (5′-AGAGTTTGATCCTGGCTCAG-3′) and 1492R (5′-TACGGCTACCTTGTTACGACTT-3′) primer pair, and the sequencing was conducted using the 785F (5′-GGATTAGATACCCTGGTA-3′) and 907R (5′-CCGTCAATTCMTTTRAGTTT-3′) primer pair. BLASTn (4) analysis of the 16S rRNA sequence revealed that this strain belongs to the genus *Pseudomonas*.

A single colony of the ABC1 strain was processed by Macrogen, Inc. (South Korea), including genomic DNA extraction, PCR amplification, purification, 20-kb SMRTbell library construction, and sequencing using the PacBio RS II platform. Sequence reads were filtered and assembled *de novo* utilizing the PacBio Hierarchical Genome Assembly Process version 3 (5) using default parameters in SMRT Portal version 2.3.0. The sequencing of the ABC1 strain produced 1,259,730,374 subread bases and 154,555 subreads with an *N*_50_ value of 11,735 bp. *De novo* genome assembly generated a unique contig of 4,035,896 bases with a 209× mean coverage. The genome was annotated with the NCBI Prokaryotic Genome Annotation Pipeline (PGAP) (6). The ABC1 strain has a GC content of 62%, with 3,555 predicted genes, 4 rRNAs, 56 tRNAs, and an average gene length of 1,040 bases. Multilocus phylogenetic analysis with concatenated sequences of 16S rRNA, *gyrB*, *rpoD*, and *rpoB*, a reliable method for species delineation and strain identification in *Pseudomonas* (7), showed that the ABC1 strain has a sequence identity of 92% with the Pseudomonas kirkiae strain 4C (8).

### Data availability.

The annotated genome was deposited in GenBank under the accession number CP058349. This project has been submitted under BioProject number PRJNA641577 and BioSample accession number SAMN15356716. The PacBio raw reads are available in the SRA under the accession number SRR12183682.

## References

[1] Peix A, Ramírez-Bahena M-H, Velázquez E 2018 The current status on the taxonomy of *Pseudomonas* revisited: an update. Infect Genet Evol 57:106–116. doi:10.1016/j.meegid.2017.10.026. 29104095

[2] Wu X, Monchy S, Taghavi S, Zhu W, Ramos J, van der Lelie D 2011 Comparative genomics and functional analysis of niche-specific adaptation in *Pseudomonas putida*. FEMS Microbiol Rev 35:299–323. doi:10.1111/j.1574-6976.2010.00249.x. 20796030 PMC3056050

[3] Jungles MK, Figueroa M, Morales N, Val del Río A, Costa RH, Campos JL, Mosquera-Corral A, Méndez R 2011 Start up of a pilot scale aerobic granular reactor for organic matter and nitrogen removal. J Chem Technol Biotechnol 86:763–768. doi:10.1002/jctb.2589.

[4] Altschul SF, Gish W, Miller W, Myers EW, Lipman DJ 1990 Basic local alignment search tool. J Mol Biol 215:403–410. doi:10.1016/S0022-2836(05)80360-2. 2231712

[5] Chin C-S, Alexander DH, Marks P, Klammer AA, Drake J, Heiner C, Clum A, Copeland A, Huddleston J, Eichler EE, Turner SW, Korlach J 2013 Nonhybrid, finished microbial genome assemblies from long-read SMRT sequencing data. Nat Methods 10:563–569. doi:10.1038/nmeth.2474. 23644548

[6] Tatusova T, DiCuccio M, Badretdin A, Chetvernin V, Nawrocki EP, Zaslavsky L, Lomsadze A, Pruitt KD, Borodovsky M, Ostell J 2016 NCBI Prokaryotic Genome Annotation Pipeline. Nucleic Acids Res 44:6614–6624. doi:10.1093/nar/gkw569. 27342282 PMC5001611

[7] Gomila M, Peña A, Mulet M, Lalucat J, García-Valdés E 2015 Phylogenomics and systematics in *Pseudomonas*. Front Microbiol 6:214. doi:10.3389/fmicb.2015.00214. 26074881 PMC4447124

[8] Bueno-Gonzalez V, Brady C, Denman S, Allainguillaume J, Arnold D 2020 *Pseudomonas kirkiae* sp. nov., a novel species isolated from oak in the United Kingdom, and phylogenetic considerations of the genera *Pseudomonas*, *Azotobacter* and *Azomonas*. Int J Syst Evol Microbiol 70:2426–2434. doi:10.1099/ijsem.0.004055. 32068524

